# The prevalence of genotypes that determine resistance to macrolides, lincosamides, and streptogramins B compared with spiramycin susceptibility among erythromycin-resistant *Staphylococcus epidermidis*

**DOI:** 10.1590/0074-02760150356

**Published:** 2016-03

**Authors:** Marek Juda, Beata Chudzik-Rzad, Anna Malm

**Affiliations:** Medical University of Lublin, Department of Pharmaceutical Microbiology, Lublin, Poland

**Keywords:** Staphylococcus epidermidis, MLS_B_ antibiotics, resistance, genotypes, spiramycin

## Abstract

Coagulase-negative staphylococci, particularly *Staphylococcus epidermidis*, can be regarded as potential reservoirs of resistance genes for pathogenic strains, e.g., *Staphylococcus aureus.* The aim of this study was to assess the prevalence of different resistance phenotypes to macrolide, lincosamide, and streptogramins B (MLSB) antibiotics among erythromycin-resistant *S. epidermidis*, together with the evaluation of genes promoting the following different types of MLSB resistance:*ermA*, *ermB*, *ermC*,*msrA*, *mphC*, and *linA/A’*. Susceptibility to spiramycin was also examined. Among 75 erythromycin-resistant*S. epidermidis* isolates, the most frequent phenotypes were macrolides and streptogramins B (MSB) and constitutive MLSB (cMLSB). Moreover, all strains with the cMLSB phenotype and the majority of inducible MLSB (iMLSB) isolates were resistant to spiramycin, whereas strains with the MSB phenotype were sensitive to this antibiotic. The D-shape zone of inhibition around the clindamycin disc near the spiramycin disc was found for some spiramycin-resistant strains with the iMLSB phenotype, suggesting an induction of resistance to clindamycin by this 16-membered macrolide. The most frequently isolated gene was *ermC*, irrespective of the MLSB resistance phenotype, whereas the most often noted gene combination was*ermC*, *mphC*, *linA/A’*. The results obtained showed that the genes responsible for different mechanisms of MLSB resistance in *S. epidermidis* generally coexist, often without the phenotypic expression of each of them.

Coagulase-negative staphylococci (CoNS), particularly *Staphylococcus epidermidis*, belong to the microbiota of human skin and the mucosal membrane of the upper respiratory tract, and they express low pathogenic potential as commensals in healthy people (Voung & Otto 2002, Otto 2009). However, they can be responsible for several serious infections in immunocompromised patients, particularly those associated with biomaterials (e.g., catheters, prosthetics etc.), leading to bacteraemia and sepsis (Ziebuhr et al. 2006,Caesy et al. 2007, Schoenfelder et al. 2010, Castro-Alarcón et al. 2011). On the other hand, as a natural part of the microflora, drug resistant strains may be selected during antibiotic therapy, which is a potential source of the resistance genes for pathogenic strains, e.g.,*Staphylococcus aureus* (Reyes et al. 2007, Otto 2013, Vitali et al. 2014).

Resistance to macrolide, lincosamide, and streptogramins B (MLS_B_ antibiotics) in staphylococci is associated with the following three mechanisms: (i) target modification, (ii) efflux pumps, and (iii) enzymatic modification of antibiotics. The first macrolide-resistant staphylococcal strains were identified in the 1950s (Roberts 2004). Currently, a large number of strains exhibit resistance to these antibiotics *via* different mechanisms. It is known that macrolide-resistant strains often exhibit co-resistance to other MLS_B_ antibiotics. The most common mechanism is the modification of ribosomes as a result of methylation of adenine within 23S rRNA ribosomal subunits by a methylase encoded by the *erm* genes (predominantly*ermC*). Conformational changes in the ribosome result in the reduced binding of all MLS_B_ antibiotics; these strains are resistant to all MLS_B_ antibiotics (the combination of quinupristin/dalfopristin loses bactericidal activity as the result of the development of resistance to quinupristin). The phenotypic expression of MLS_B_ resistance can be either inducible (iMLS_B_) (generally induced by 14 and 15-membered macrolides) or constitutive (cMLS_B_) (Weisblum 1995). The active efflux of antibiotics is mediated by *msr* genes (mainly*msrA*) and is responsible for resistance only to 14 and 15-membered macrolides and streptogramins B (MS_B_) phenotype (Reynolds et al. 2003). The third mechanism of resistance is based on the production of antibiotic-inactivating enzymes (e. g., phosphorylase encoded by*mph* or *lin*, the gene responsible for inactivation of lincosamides) (Chesneau et al. 2007, Achard et al. 2008).

The aim of this study was to assess the prevalence of different MLS_B_resistance phenotypes among *S. epidermidis*, together with the evaluation of genes responsible for target modification (*ermA*,*ermB*, *ermC*), antibiotic efflux (*msrA*) or antibiotic inactivation (*mphC*,*linA/A’*). The evaluation of susceptibility to the 16-membered macrolide spiramycin was also performed.

This paper was developed using the equipment purchased within agreement POPW.01.03.00-06-010/09-00 Operational Program Development of Eastern Poland 2007-2013, Priority Axis I, Modern Economy, Operations 1.3. Innovations Promotion.

## SUBJECTS, MATERIALS AND METHODS

*Bacterial strains* - A total of 197 strains of *S. epidermidis* were obtained from the mucosal membranes of the upper respiratory tracts of patients with nonsmall cell lung cancer who underwent hospitalisation. Nasal and pharyngeal swabs were obtained on the second day of the patients’ stays at the hospital. Among the strains, resistance to erythromycin was detected in 75 isolates.

*Isolation and identification* - Isolation and identification of bacterial strains were performed using routine microbiological tests. The following tests were used in the identification of CoNS: the coagulase test tube using rabbit plasma (Biomed, Poland) and API Staph strips (bioMérieux, France).

*Identification of resistance to MLS*_*B*_ *antibiotics* - Susceptibility to MLS_B_ antibiotics, including the detection of resistance mechanisms, was based on the D-test according to European Centre for Disease Prevention and Control (EUCAST) recommendations. In addition, disks containing lincomycin (15 mg) were used to identify the L-phenotype. Moreover, for detection of the effects of spiramycin on clindamycin susceptibility, discs containing spiramycin (100 mg) were applied next to clindamycin (2 mg).

*Determination of minimal inhibitory concentrations (MICs) to spiramycin* - Detection of MICs to spiramycin was based on EUCAST recommendations using the double broth dilution method. In the absence of breakpoints for spiramycin in EUCAST, only the MICs were evaluated without grouping the strains as susceptible or resistant.

*Isolation of bacterial DNA* - The DNA Genomic Mini Kit (A&A Biotechnology, Poland) was used to isolate *S. epidermidis* DNA according to the manufacturer’s guidelines.

*Identification of genes by polymerase chain reaction (PCR)* - The sequences of the primers and the conditions of the PCR reactions are presented inTable I. For the PCR reactions, PCR REDTaq^®^ Ready Mix^TM^ PCR Mix with MgCl_2_(Sigma-Aldrich, USA) was used. The final volume of each PCR reaction was 25 ml and contained 12.5 ml of REDTaq Ready Mix, 1 ml of each forward and reverse primer (concentration between 0.1-1.0 mM), 1 ml of DNA (50-200 ng), and 9 ml of water. The reactions were performed using a Whatman Biometra thermocycler, whereas the PCR products were subjected to agarose gel electrophoresis (2% agarose, 1xTRIS-acetate-EDTA, 120 mV, 40 min). The gels were stained with ethidium bromide and the PCR products were visualised using a Wilbert Lambert transilluminator and compared with molecular size markers [Gene Ruler^TM^ 100 bp DNA Ladder (Fermentas, Thermo Scientific, USA)].

**TABLE I:** Primers sequence, thermal cycling profile, and size of amplified polymerase chain reaction (PCR) fragment in each PCR reaction in the detection of genes of *Staphylococcus epidermidis*resistant to erythromycin*a*

Gene	Primers sequence	PCR conditions	PCR fragment size (bp)
*ermA*	5’-TCTAAAAAGCATGTAAAAGAA-3’ 5’-CTTCGATAGTTTATTAATATTAGT-3’	35 (30 s at 94ºC, 1 min at 48ºC, 2 min at 72ºC)	645
*ermB*	5’-GAAAAGGTACTCAACCAAATA-3’ 5’-AGTAACGGTACTTAAATTGTTTAC-3’	35 (30 s at 94ºC, 30 s at 50ºC, 2 min at 72ºC)	639
*ermC*	5’-AGTACAGAGGTGTAATTTCG-3’ 5’-AATTCCTGCATGTTTTAAGG-3’	35 (55 s at 94ºC, 1 min at 53ºC, 1 min at 72ºC)	642
*msrA*	5’-GGCACAATAAGAGTGTTTAAAGG-3’ 5’-AAGTTATATCATGAATAGATTGTCCTGTT-3’	25 (1 min at 94ºC, 1 min at 50ºC, 90 s at 72ºC)	399
*mphC*	5’-GAGACTACCAGACCTGACG-3’ 5’-CATACGCCGATTCTCCTGAT-3’	35 (1 min at 94ºC, 1 min at 59ºC, 1 min at 72ºC)	530
*linA/A’*	5’-GGTGGCTGGGGGGTAGATGTATTAACTGG-3’ 5’-GCTTCTTTTGAAATACATGGTATTTTTCGATC-3’	30 (30 s at 94ºC, 30 s at 57ºC, 1 min at 72ºC)	323

*a*: Sutcliffe et al. (1996) and Lina et al. (1999).

*Ethics* - The study design and protocols were approved by the Ethical Committee of the Medical University of Lublin (KE-0254/75/2011).

## RESULTS

The 75 *S. epidermidis* isolates expressed resistance to erythromycin with the following mechanisms of resistance: 27 (36%) strains exhibited cMLS_B_ resistance, 14 (18.7%) strains exhibited iMLS_B_resistance, and 34 (45.3%) strains exhibited MS_B_ resistance (Figure). Twenty-five isolates exhibited L-phenotypes and were determined to be either resistant to only lincomycin (24 strains) or resistant to lincomycin and clindamycin (1 strain).

**Figure:**
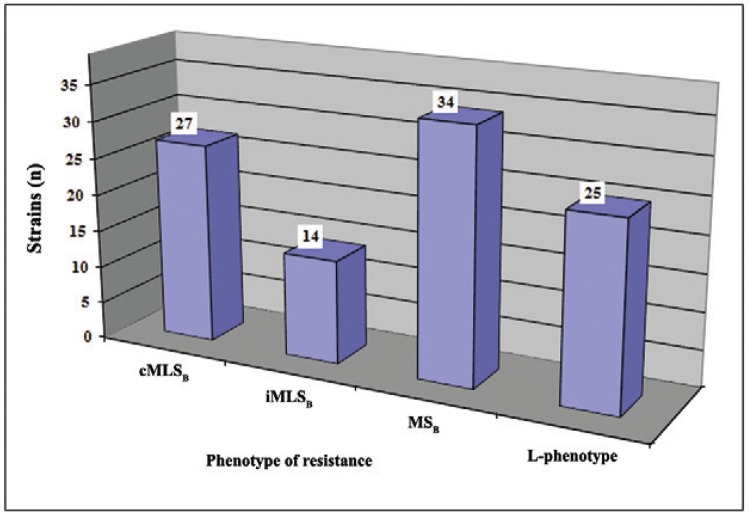
The prevalence of different mechanisms of resistance to macrolide, lincosamide, and streptogramins B (MLSB) antibiotics among erythromycin-resistant *Staphylococcus epidermidis*. cMLSB: constitutive resistance to MLSB antibiotics; iMLSB: inducible resistance to MLSB antibiotics; MSB: resistance of MSB type.

The MICs of spiramycin among erythromycin-resistant *S. epidermidis*were evaluated as follows: > 128 mg/L for all cMLS_B_ strains, from 4-> 128 mg/L for iMLS_B_ strains, and from 1-4 mg/L for strains exhibiting the MS_B_ phenotype. The MIC_50_ and MIC_90_values were also calculated. Strains with cMLS_B_ and iMLS_B_phenotypes exhibited MIC_50_ and MIC_90_ values > 128 mg/L, whereas the MIC_50_ and MIC_90_ values for the MS_B_strains were determined to 4 mg/L (Table II). Moreover, for the 11 (78.6%) strains exhibiting iMLS_B_ phenotypes, the noninhibition zone around the spiramycin disc was found together with a D-shaped zone around the clindamycin disk.

**TABLE II:** The minimal inhibitory concentrations (MICs) to spiramycin among erythromycin-resistant *Staphylococcus epidermidis*

mg/L	iMLS_B_	cMLS_B_	MS_B_
MIC range	4-> 128	> 128	1-4
MIC_50_	> 128	> 128	4
MIC_90_	> 128	> 128	4

cMLS_B_: constitutive resistance to macrolide, lincosamide, and streptogramins B (MLS_B_) antibiotics; iMLS_B_: inducible resistance to MLS_B_antibiotics; MS_B_: resistance of MS_B_ type.

As shown in Table III, among the strains with cMLS_B_ resistance, the predominant genes were *ermC*and *mphC* in 23 (85.2%) and 24 (88.9%) strains, respectively.*linA/A*’ was found to occur in 14 (51.8%) strains. The presence of other genes (e.g., *ermA* and *ermB*) was detected in a few strains; two strains did not possess any of the *erm* genes. The isolates with iMLS_B_ possessed the following genes:*ermC* - 14 (100%) strains, *msrA* - 7 (50%) strains, *mphC* - 13 (92.9%) strains, and *linA/A’* - 10 (71.4%) strains; *ermA* and *ermB* were not detected. The strains exhibiting MS_B_ resistance were found to possess the following genes: *ermC* in 20 (58.8%) strains, *msrA*in 32 (94.1%) strains, *mphC* in 33 (97.1%) strains, and*linA/A’* in 24 (70.6%) strains; these strains did not carry*ermA* or *ermB*. The strains exhibiting L-phenotypes contained *linA/A’* in 24 (96%) strains,*mphC* in 23 (92%) strains, and *ermC* in 24 (96%) strains. *ermA*, *ermB*, and *msrA*were not detected in the isolates with L-phenotypes. One strain did not carry any of the evaluated genes.

**TABLE III:** The prevalence of genes responsible for resistance to macrolide, lincosamide, and streptogramins B (MLSB) antibiotics among erythromycin-resistant *Staphylococcus epidermidis*

Gene	Phenotypes n (%)			

	cMLS_B_ (n = 27)	iMLS_B_ (n = 14)	MS_B_ (n = 34)	L-phenotype (n = 25)
*ermA*	4 (14.8)	0 (0)	0 (0)	0 (0)
*ermB*	1 (3.7)	0 (0)	0 (0)	0 (0)
*ermC*	23 (85.2)	14 (100)	20 (58.8)	24 (96)
*msrA*	5 (18.5)	7 (50)	32 (94.1)	0 (0)
*mphC*	24 (88.9)	13 (92.9)	33 (97.1)	23 (92)
*linA/A’*	14 (51.8)	10 (71.4)	24 (70.6)	24 (96)

cMLS_B_: constitutive resistance to MLS_B_antibiotics; iMLS_B_: inducible resistance to MLS_B_ antibiotics; MS_B_: resistance of MS_B_ type.

Table IV shows the combination of genes responsible for resistance to MLS_B_ antibiotics among staphylococci. In isolates exhibiting cMLS_B_ resistance, 11 different combinations were detected. The most frequent gene combination was *ermC*,*mphC*, and *linA/A’*, which was found in 10 (37%) strains. Among the strains exhibiting iMLS_B_ resistance, four gene combinations were evaluated. The most frequent combinations contained the following genes: *ermC*, *mphC*, and *linA/A’* in five (35.7%) isolates and *ermC*, *msrA*,*mphC*, and *linA/A’*, also in five (35.7%) isolates. The MS_B_-positive strains contained six different gene combinations in three major groups: *ermC*, *msrA*,*mphC*, and *linA/A’* in 14 (41.2%) strains;*msrA*, *mphC*, and *linA/A’* in nine (26.5%) strains, and *ermC*, *msrA*, and*mphC* in six (17.6%) strains. In the isolates with L-phenotypes, the most significant three-gene combination was *ermC*,*mphC*, and *linA/A’* in 21 (84%) strains.

**TABLE IV:** The prevalence of gene combinations responsible for resistance to macrolide, lincosamide, and streptogramins B (MLSB) antibiotics among erythromycin-resistant *Staphylococcus epidermidis*

Gene combinations	Phenotypes n (%)			

	cMLS_B_ (n = 27)	iMLS_B_ (n = 14)	MS_B_ (n = 34)	L-phenotype (n = 25)
*ermC*	1 (3.7)	1 (7.1)	0 (0)	0 (0)
*mphC*	0 (0)	0 (0)	2 (5.9)	0 (0)
*ermC*, *mphC*	4 (14.8)	3 (21.4)	0 (0)	0 (0)
*ermB*, *mphC*	1 (3.7)	0 (0)	0 (0)	0 (0)
*ermC*, *linA/A’*	1 (3.7)	0 (0)	0 (0)	2 (8)
*ermA*, *mphC*	1 (3.7)	0 (0)	0 (0)	0 (0)
*msrA*, *mphC*	0 (0)	0 (0)	2 (5.9)	0 (0)
*msrA*, *linA/A’*	0 (0)	0 (0)	1 (2.9)	0 (0)
*mphC*, *linA/A’*	0 (0)	0 (0)	0 (0)	1 (4)
*ermC*, *msrA*,*mphC*	3 (11.1)	0 (0)	6 (17.6)	0 (0)
*ermC*, *mphC*,*linA/A’*	10 (37)	5 (35.7)	0 (0)	21 (84)
*msrA*, *mphC*,*linA/A’*	1 (3.7)	0 (0)	9 (26.5)	0 (0)
*ermA*, *ermC*,*mphC*	2 (7.4)	0 (0)	0 (0)	0 (0)
*ermC*, *msrA*,*mphC*, *linA/A’*	1 (3.7)	5 (35.7)	14 (41.2)	0 (0)
*ermA*, *ermC*,*mphC*, *linA/A’*	1 (3.7)	0 (0)	0 (0)	0 (0)
Without genes	1 (3.7)	0 (0)	0 (0)	1 (4)

cMLS_B_: constitutive resistance to MLS_B_antibiotics; iMLS_B_: inducible resistance to MLS_B_ antibiotics; MS_B_: resistance of MS_B_ type.

## DISCUSSION

CoNS are potential reservoirs of antibiotic resistance genes, which can be transferred to *S. aureus* not only in vitro but also in vivo (Reyes et al. 2007, Otto 2013). Erythromycin resistance among CoNS was previously reported to result from a methylase encoded by different *erm* family genes that can be horizontally transferred to recipient strains (Zmantar et al. 2011, Vitali et al. 2014). Hence, surveillance of erythromycin resistance and MLS_B_ resistance in CoNS at phenotypic and genetic levels can provide important information regarding their current epidemiology.

Among the *S. epidermidis* strains studied, the most frequently identified gene in strains exhibiting both cMLS_B_ and iMLS_B_phenotypes was *ermC*, which is consistent with previous reports (Reyes et al. 2007, Gherardi et al. 2009, Coutinho et al. 2010, Bouchami et al. 2011,Brzychczy-Wloch et al. 2013, Heb & Gallert 2014). Only a few *S. epidermidis* exhibiting cMLS_B_ phenotypes possessed*ermA* and/or *ermB.* Similar data have been previously reported (Bouchami et al. 2011,Teodoro et al. 2012, Szczuka et al. 2016). Moreover, the presence of other *erm* genes (e.g., *ermF)* has been rarely detected in *Staphylococcus* spp (Roberts 2004). Notably, the distribution of *erm* genes depends on the bacterial species. For example, *ermA* is more characteristic of *S. aureus,* whereas *ermB* is more characteristic of beta-haemolytic streptococci (Roberts 2004, Buter et al. 2010,Meehan et al. 2014, Vitali et al. 2014). Moreover, among CoNS, the type of*erm* gene also depends on the geographical region of their isolation. For example, *ermC* was previously detected in 50% of the strains exhibiting MLS_B_ resistance in Great Britain, whereas it was detected 90% of those in Denmark (Lim et al. 2002, Gatermann et al. 2007, Cetin et al. 2010, Bouchami et al. 2011) and in Mexico, *ermA* was reported as predominant in *S. epidermidis* (Castro-Alarcón et al. 2011).

The MS_B_ *S. epidermidis* isolates examined contained an*msrA* gene encoding an ATP-dependent efflux pump, which actively removes 14-,15-membered MS_B_. The MS_B_ phenotype observed in*msrA*-negative *S. epidermidis* strains may be the result of the presence of *mphC*, which encodes for a macrolide-modifying enzyme (Gatermann et al. 2007), thereby resulting in a “false-positive” MS_B_phenotype.

All *S. epidermidis* isolates with L-phenotypes generally contained the *linA/A’* gene. Data from Novotna et al. (2005, 2007) also indicated a connection between the presence of the *linA/A’* gene and resistance to only lincomycin among staphylococci. The *S. epidermidis* strains studied exhibited resistance to lincomycin, but susceptibility to clindamycin as a result of increased enzyme affinity for lincomycin (Achard et al. 2005). Resistance both to lincomycin and clindamycin may be a consequence of the presence of other*lin* family genes or*vga(A)*_*LC*_, which encodes a “new” variant of the SgA protein that is responsible for cross-resistance to streptogramins A and all lincosamides (Novotna & Janata 2006).

Among the iMLS_B_ and cMLS_B_ *S. epidermidis*strains, the *erm* genes do not exist separately, but in combination with others (predominantly with *mphC*). Notably, other*erm* genes (e.g., *ermF*), which are rarely detected in *Sta- phylococcus* spp, may encode both the inducible or constitutive MLS_B_ phenotypes (Roberts 2004). In MS_B_-positive *S. epidermidis*strains, the *msrA* genes predominantly coexist with*ermC*, *mphC*, and *linA/A’*, and the coexistence of *msrA* and *ermC* has also been previously reported (Roberts 2004, Novotna et al. 2007, Wang et al. 2008, Teodoro et al. 2012). Moreover, the presence of the *linA/A’* gene in*msrA*-positive strains results in resistance to lincomycin. The*S. epidermidis* strains exhibiting L-phenotypes correlated with the presence of the *linA/A’* gene in most of the strains that also contained the *ermC* and *mphC* genes, whereas those strains did not contain the *msrA* gene. Notably, the*ermC* genes were also detected in both of the MS_B_ and L-phenotype *S. epidermidis* strains - but without its expression - suggesting a defect in *ermC* expression.

Previous studies have reported (Leclercq 2002,Coutinho et al. 2010) that 16-membered macrolides (e.g., spiramycin) are not inducers of MLS_B_ resistance in staphylococci. According to our data, spiramycin is able to induce resistance to clindamycin among the iMLS_B_ *S. epidermidis* isolates examined. Moreover, iMLS_B_ *S. epidermidis* strains, which contain *ermC*, exhibited resistance to spiramycin in vitro. These observations contradict previous reports that 16-membered macrolides remain active against staphylococci that exhibit iMLS_B_ phenotypes (Leclercq 2002, Szczuka et al. 2016). Notably, resistance to spiramycin appears to be characteristic of iMLS_B_ streptococci containing *ermB*(Leclercq 2002, Acikgoz et al. 2003).

The diversity of genes involved in different mechanisms that are responsible for the resistance of *S. epidermidis* to MLS_B_ antibiotics suggests that the insensitivity of CoNS strains to these antibacterial drugs is not necessarily a unidirectional process and that the coexistence of various genes may influence the nature of their resistance.
